# BcpLH organizes a specific subset of microRNAs to form a leafy head in Chinese cabbage (*Brassica rapa* ssp. *pekinensis*)

**DOI:** 10.1038/s41438-019-0222-7

**Published:** 2020-01-01

**Authors:** Wenqing Ren, Feijie Wu, Jinjuan Bai, Xiaorong Li, Xi Yang, Wanxin Xue, Heng Liu, Yuke He

**Affiliations:** 1 National Laboratory of Plant Molecular Genetics, Shanghai Institute of Plant Physiology and Ecology, Chinese Academy of Sciences, Fenglin Road 300, Shanghai, 200032 China; 2 Graduate School of the Chinese Academy of Sciences, Shanghai, 200032 China; 3 South Subtropical Crop Research Institute, Chinese Academy of Tropical Agricultural Sciences, Ministry of Agriculture, Zhanjiang, Guangdong, China

**Keywords:** Non-model organisms, Leaf development, DNA replication, Non-coding RNAs

## Abstract

*HYL1* (*HYPONASTIC LEAVES 1*) in *Arabidopsis thaliana* encodes a double-stranded RNA-binding protein needed for proper miRNA maturation, and its null mutant *hyl1* shows a typical leaf-incurvature phenotype. In Chinese cabbage, *BcpLH* (*Brassica rapa ssp. pekinensis LEAFY HEADS*), a close homolog of *HYL1*, is differentially expressed in juvenile leaves, which are flat, and in adult leaves, which display extreme incurvature. BcpLH lacks protein–protein interaction domains and is much shorter than HYL1. To test whether *BcpLH* is associated with defects in microRNA (miRNA) biogenesis and leaf flatness, we enhanced and repressed the activity of *BcpLH* by transgenics and investigated *BcpLH*-dependent miRNAs and plant morphology. BcpLH promoted miRNA biogenesis by the proper processing of primary miRNAs. *BcpLH* downregulation via antisense decreased a specific subset of miRNAs and increased the activities of their target genes, causing upward curvature of rosette leaves and early leaf incurvature, concurrent with the enlargement, earliness, and round-to-oval shape transition of leafy heads. Moreover, BcpLH-dependent miRNAs in Chinese cabbage are not the same as HYL1-dependent miRNAs in Arabidopsis. We suggest that *BcpLH* controls a specific subset of miRNAs in Chinese cabbage and coordinates the direction, extent, and timing of leaf curvature during head formation in *Brassica rapa*.

## Introduction

Leafy heads are types of agricultural product composed of numerous incurved leaves. Crop species with leafy heads include Chinese cabbage (*Brassica rapa* ssp*. pekinensis*, syn. *Brassica campestris* ssp. *pekinensis*), cabbage (*B. oleracea* var*. capitata*), brussels sprouts (*B. oleracea* var*. gemmifera*), and lettuce (*Lactuca sativa*). Unlike the grains of corn, rice, and wheat, which provide starch and proteins for food, leafy heads supply mineral nutrients, crude fiber, and vitamins for health. The vegetative development of these crop species is divided into seedling, rosette, folding, and heading stages. The seedling and rosette leaves perform normal photosynthesis, whereas the head leaves serve as nutrient storage organs. The flatness of rosette leaves and the proper incurvature of heading leaves are essential for the high yield and quality of leafy heads. However, the genetic basis underlying leaf incurvature and head formation is unclear.

Leaf curvature is determined by leaf morphogenesis. In particular, the roles of adaxial–abaxial polarity, cell division, phase transition, and the genesis of leaf formation have long been a focus of studies^1,2^. The flatness of leaves can be described in terms of Gaussian curvature, in which a flat surface grows isotropically; for example, a uniformly expanding disc maintains zero Gaussian curvature^3^. Although leaves of many plant species have approximately zero Gaussian curvature, there are many more ways for a leaf to adopt negative or positive curvature than zero curvature for natural variation. Several transcription factors responsible for leaf adaxial–abaxial polarity have been shown to participate in the establishment of leaf curvature. The adaxial side is specified by the activity of members of the class III Homeodomain Leucine Zipper (HD-ZIP III) family of transcription factors. HD-ZIP III genes are targets of miR165/6. Of these, *PHABULOSA* (*PHB*), *PHAVOLUTA* (*PHV*), and *REVOLUTA* (*REV*) act redundantly to promote the adaxial cell fates of leaf primordia^4–7^. Dominant gain-of-function mutations in these transcription factors have been characterized that cause an expanded expression domain, promoting the adaxial growth of leaves^4,7^. *ATHB8* (*HB-8*) and *CORONA* (*CNA*) play antagonistic roles against *REV* in certain tissues while performing overlapping functions with those of REV in other tissues^4^. Loss of function of the HD-ZIPIII gene results in abaxialized organs^6,8–10^. Members of the miR319a-targeted *TEOSINTE BRANCHED1/CYCLOIDIA/PCF* (*TCP*) gene family function in the maintenance of the normal shape and flatness of leaves via arrested cell division at the front of leaves^3^. miR156-targeted *SPL* genes control the transition of leaves from the juvenile to adult stage by the mediation of morphological and physiological changes^11,12^. In Chinese cabbage, miR319a modulates the head shape of Chinese cabbage by differentially arresting cell division in leaf regions^13^. The silencing of the miR156-targeted *SPL* genes promotes early leaf incurvature and heading^14^.

MiRNAs and their targets have been shown to function in many plant development processes and to be involved in protein processing. HYL1 has been verified to participate in the biogenesis of miRNAs in combination with DICER-LIKE1 (DCL1) and SERRATE (SE)^15,16^. As a result of a reduction in miRNAs, plants with the *hyl1* null allele exhibit multiple phenotypic abnormalities, such as leaf hyponasty, delayed flowering, altered root gravity responses, and altered responses to hormones^17,18^. The N-terminal region of HYL1, which has two tandem dsRBD domains alone, is adequate to completely rescue the phenotype of *hyl1* mutant^19^.

The formation of a leafy head is a multitrait. The size, shape, weight, and compactness of leafy head and heading time are under the control of different genetic loci^20^. All plants with leafy heads undergo leaf curvature transitions from downward to inward. Leaf incurvature at late developmental stages is essential for the high yield and quality of leafy heads. In 2000, we reported that the gene *Brassica rapa ssp. pekinensis LEAFY HEADS* (*BcpLH*) was isolated by differential hybridization of cDNA libraries using flat rosette and upwardly curved folding leaves of Chinese cabbage^21^. To determine whether *BcpLH* functions in leaf curvature, we investigated *BcpLH*-regulated miRNAs and miRNA-targeted genes through the overexpression or silencing of *BcpLH*. We found that *BcpLH* controlled the timing of leaf curvature and leafy head formation through integration of some important miRNAs.

## Results

### *BcpLH* is downregulated at the folding stage of Chinese cabbage

**Fig. 1:**
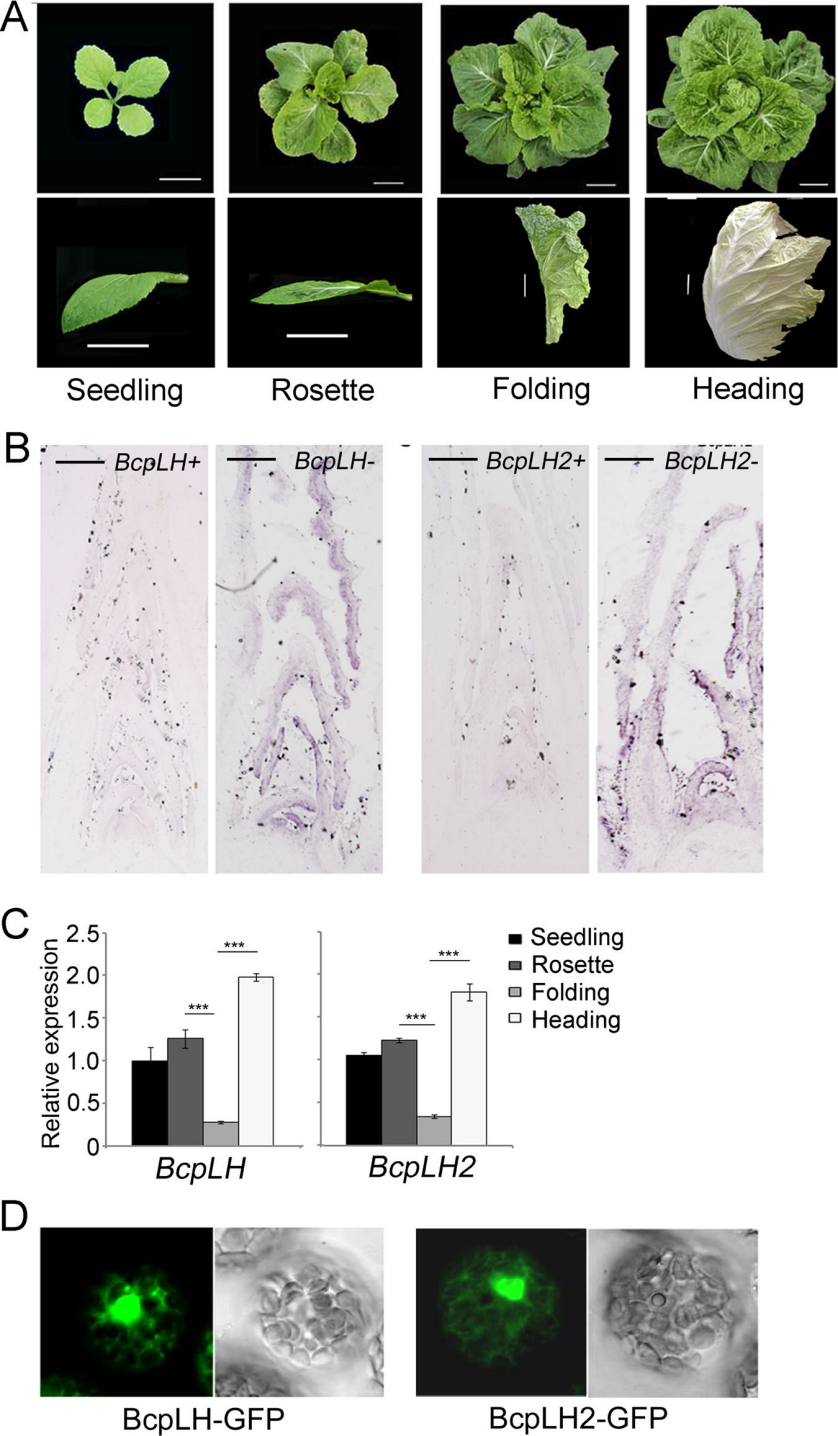
Expression patterns of BcpLH and BcpLH2 in Chinese cabbage. **a** The stages (up) and leaf shapes (bottom) of Chinese cabbage (*Brassica rapa ssp. pekinensis*); bar = 5 cm. **b** In situ hybridization of BcpLH (left) and BcpLH2 (right) in the meristem of 20-day-old Chinese cabbage. BcpLH+, sense probe of BcpLH; BcpLH-, antisense probe of BcpLH; BcpLH2+, sense probe of BcpLH2; BcpLH2-, antisense probe of BcpLH2. **c** Real-time PCR for the expression of BcpLH and BcpLH2 in 1–2 cm long developing leaves from the tip of Chinese cabbage at four stages. ACTIN expression was used as an internal control. The error bars represent the SDs calculated from three biological replicates, each of which consisted of three technical replicates. *p* < 0.01, ***. **d** Subcellular localization of BcpLH and BcpLH2 with GFP under the 35S promoter by instantaneous transformation to protoplasts of Chinese cabbage leaves.

Chinese cabbage plants are characterized by downward-curving leaves at the seedling stage, flat leaves at the rosette stage, upward-curving leaves at the folding stage and inward-curving leaves at the heading stage (Fig. 1a). *BcpLH* isolated by differential hybridization between rosette and folding leaves was considered to contribute to heading. Genomic sequencing of *B. rapa* revealed another copy of *BcpLH*, which we named *BcpLH2*. The amino acid sequences of the two BcpLH proteins are highly identical (96%), which indicated that BcpLH and BcpLH2 may function redundantly in *B. rapa*.

To examine the contribution of *BcpLH*s in Chinese cabbage, we measured the temporal and spatial expression patterns of the *BcpLH* genes. We isolated RNA samples from the shoot tips of plants at the four developmental stages. Real-time PCR showed that the expression levels of both *BcpLH* or *BcpLH2* increased progressively from the seedling stage, during the rosette stage and to the heading stage, while they were downregulated at the folding stage (Fig. 1c). This result was consistent with that of the differential hybridization, which indicated that *BcpLH* functions at the key folding stage. In situ hybridization demonstrated that both *BcpLH* and *BcpLH2* were expressed mainly in the shoot apical meristems and developing leaves. The difference is that, compared with *BcpLH2* expression, *BcpLH* expression in developing leaves was more preferential in the adaxial region than in the abaxial regions (Fig. 1b). *BcpLH2* was expressed mainly in the shoot apical meristem and tips of developing leaves, whereas *BcpLH* was expressed preferentially in the adaxial regions of developing leaves (Fig. 1b).

To investigate the subcellular localization of BcpLH, we fused GFP with BcpLH and performed a transient expression of *p35S:BcpLH-GFP* and *p35S:BcpLH2-GFP* in leaf protoplasts of Chinese cabbage. Subcellular fluorescence showed that BcpLH and BcpLH2 were localized simultaneously in the nucleus and cytoplasm (Fig. 1d).

### The knockdown of *BcpLH* altered the timing of leaf curvature and leafy head formation

**Fig. 2:**
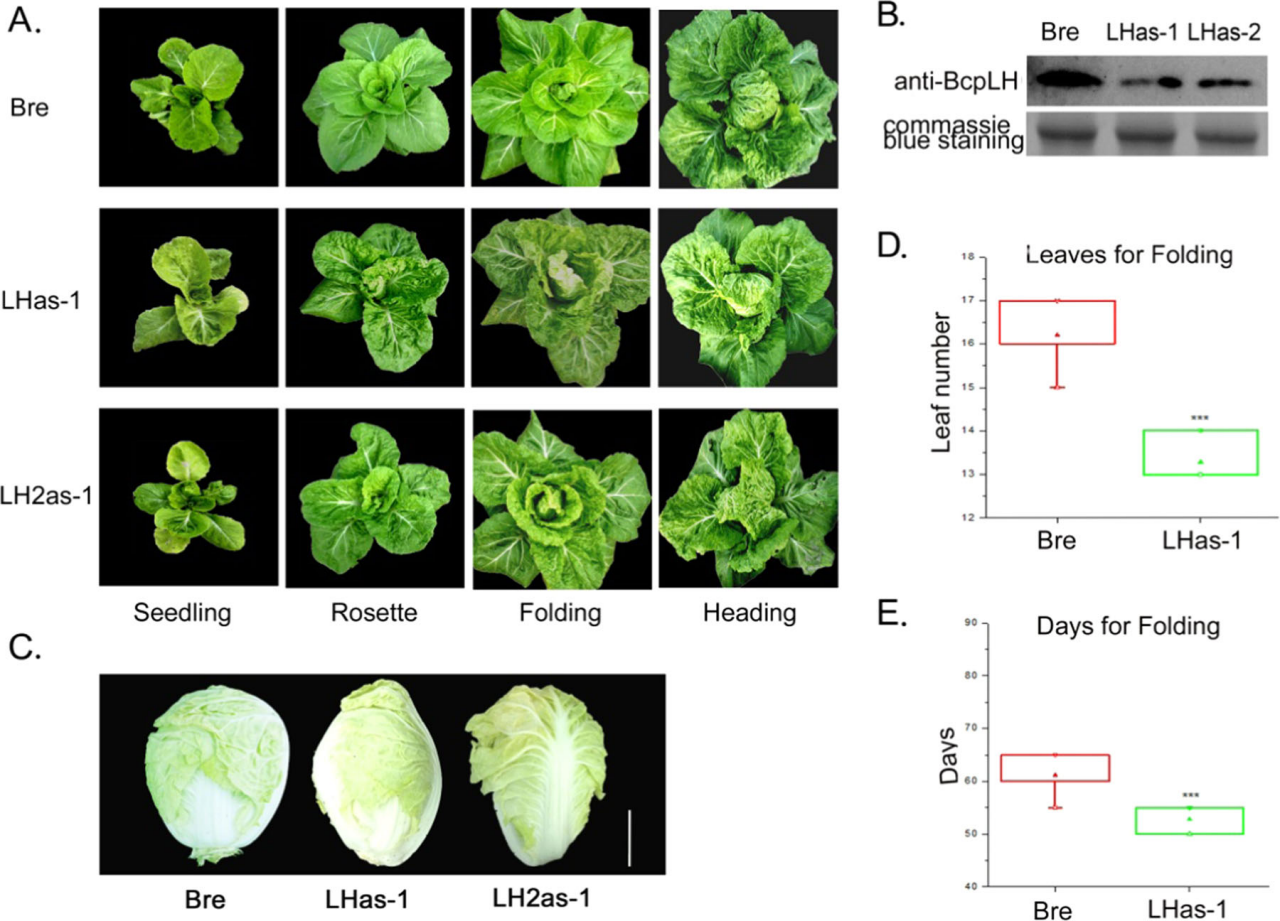
BcpLH controls leaf curvature and heading characteristics of Chinese cabbage. **a** Phenotype of BcpLH and BcpLH2 antisense transgenic plants at four stages in the field. LHas-1, pAA6:BcpLH antisense/Bre. LH2as-1, pAA6:BcpLH2 antisense/Bre. **b** Western blot showing the BcpLH protein levels in BcpLH antisense transgenic plants, with Coomassie staining used as a control. LHas-1 and LHas-2 represent BcpLH antisense transgenic lines 1 and 2, respectively. **c** Heads of BcpLH and BcpLH2 antisense transgenic plants in the field. Bar = 10 cm. **d** Number of leaves and (**e**) days at the folding stage of Bre and LHas-1 plants, analyzed by Minitab. *n* = 25; *p* < 0.01, ***.

Considering the special expression pattern of *BcpLH*, we hypothesized that *BcpLH* plays a key role in the heading of Chinese cabbage. First, we overexpressed *BcpLH* in Chinese cabbage under the control of the AA6 promoter using *in planta* transformation via the vernalization-infiltration method^22^. The phenotype of the transgenic plants did not differ from that of the wild type, even though *BcpLH* mRNA and protein levels markedly increased (Supplementary Fig. 1). To determine the physiological roles of *BcpLH* and *BcpLH2*, we cloned Bre antisense sequences of *BcpLH* and *BcpLH2* and inserted them into binary vectors under the control of the AA6 promoter and then transferred those constructs into Bre plants of Chinese cabbage plants that produced a round head. We named the two *pAA6:BcpLH* antisense transgenic lines LHas-1 and LHas-2 and the two *pAA6:BcpLH2* antisense lines LH2as-1 and LH2as-2 (Fig. 2a). To confirm the knockdown of *BcpLH* in the transgenic plants with antisense *BcpLH*, we isolated protein samples from developing leaves (1 cm long) of the LHas-1 plants at the rosette stage. Western blotting showed that the amount of BcpLH protein was reduced in the LHas-1 cells (Fig. 2b), revealing that antisense BcpLH specifically reduced the accumulation of BcpLH proteins. In the field, compared with the wild-type plants, which had flat leaves and a round head, the four transgenic lines showed more crinkly leaves, an earlier heading time and a head shape that transitioned from round to cylindrical (Fig. 2a). The 13th leaf of the Bre plants was flat at the rosette stage, and the 16th–17th leaves began to fold (Fig. 2a, d). By contrast, the 13th leaves of the LHas-1 lines were curved inward, with crinkles and more bulges, exhibiting the properties of leaves at the folding and heading stages (Fig. 2a, d). Compared to that of the wild type, the first day with upward curvature in the LHas-1 plants was 10 days earlier (Table 1), and the first leaf with upward curvature was earlier (by 3 leaves); in addition, in the latter, the juvenile phase (seedling stage) was 4 days shorter, and there was 1 fewer leaf in the juvenile phase (Fig. 2e). As a result, the timing of the upward and inward curvature of the LHas-1 leaves occurred much earlier than did that of the wild types, leading to early heading, and the number of incurved leaves increased, resulting in taller, heavier, and larger heads.

**Table 1:** Times at which leaf curvature begins and head formation occurs in the transgenic plants harboring antisense *BcpLH* (LHas-1).

Times	WT	LHas-1
First day with downward-curving leaves	10	10
First day with flat leaves	40.6 ± 4.44	37.3 ± 3.07
First day with upward-curving leaves	57.8 ± 3.56	47.0 ± 3.16
First day with inward-curving leaves	61.2 ± 3.61	52.8 ± 2.53
First day with a mature head	74.6 ± 3.12	63.7 ± 2.23
First downward-curving leaf	1	1
First fat leaf	6.92 ± 0.49	6.2 ± 0.62
First upward-curving leaf	14.8 ± 0.82	11.0 ± 1.21
First inward-curving leaf	16.2 ± 0.76	13.3 ± 0.45
First mature leaf	21.0 ± 0.73	17.6 ± 0.65
Days of the juvenile phase	41.3 ± 4.41	37.2 ± 3.07
Days of the early adult phase	20.6 ± 3.67	15.5 ± 2.29
Days of the late adult phase	12.8 ± 2.29	10.9 ± 2.02
Days of the heading stage	29.7 ± 3.05	28.3 ± 1.44
Leaves during the juvenile phase	6.9 ± 0.49	6.2 ± 0.62
Leaves during the early adult phase	9.3 ± 0.74	7.1 ± 0.60
Leaves during the late adult phase	4.8 ± 1.05	4.3 ± 0.75
Leaves during the heading stage	38.7 ± 2.18	43.4 ± 4.83
Head compactness	+	++
Head shape	Round	Oval
Head diameter (cm)	13.4 ± 1.3	13 ± 0.7
Head height (cm)	18.5 ± 1.2	21.0 ± 0.8
Head weight (kg)	0.65 ± 0.2	0.75 ± 0.2

The seeds were sown in pots and grown at 22 °C in a SIPPE phytotron. The seedlings were transplanted into the field on August 24, 2015, at a SIPPE farm station. More than 20 plants were used for each measurement. The number of days was recorded from the first day after germination, while the number of leaves from the first primary leaf was recorded. The data are presented as the mean of 20 plants

*ND* not detected

While the rosette leaves of the wild-type plants were flat, those of the LHas-1 and LHas-2 plants wrinkled, with bugles and wavy margins. Compared to that in the wild-type leaves, the longitudinal curvature of the top regions in LHas-1 leaves became weaker, causing the head shape to transition from the round to oval (Fig. 2a, c; Supplementary Table 1). While the leafy heads of the LHas-1 and LHas-2 plants were round shaped, those of the LHas-1 and LHas-2 plants were oval, apparently due to the constriction of the top regions of head leaves.

### *BcpLH* is the homologous gene of *HYL1* and rescues the phenotype of *hyl1* plants

Knockdown of *BcpLH* and *BcpLH2* affected the heading of Chinese cabbage, so the next problem was to determine how *BcpLH* and *BcpLH2* regulate heading. First, the amino acid sequences composing BcpLH and BcpLH2 were queried via BLAST. We found that BcpLH and BcpLH shared high identity, approximately 78%, with the two dsRNA-binding domains of HYL1 in *Arabidopsis* (Supplementary Fig. 2A). Compared with HYL1, both the BcpLH and BcpLH2 proteins have two conserved dsRNA-binding domains but lack the long C-terminal fragments containing a putative protein–protein interaction (PPI) domain. A phylogenetic tree of AtDRBs and BcpDRBs was constructed and showed that *BcpLH* and *BcpLH2* were the definite orthologous genes of *HYL1* in Chinese cabbage (Supplementary Fig. 2B).

**Fig. 3:**
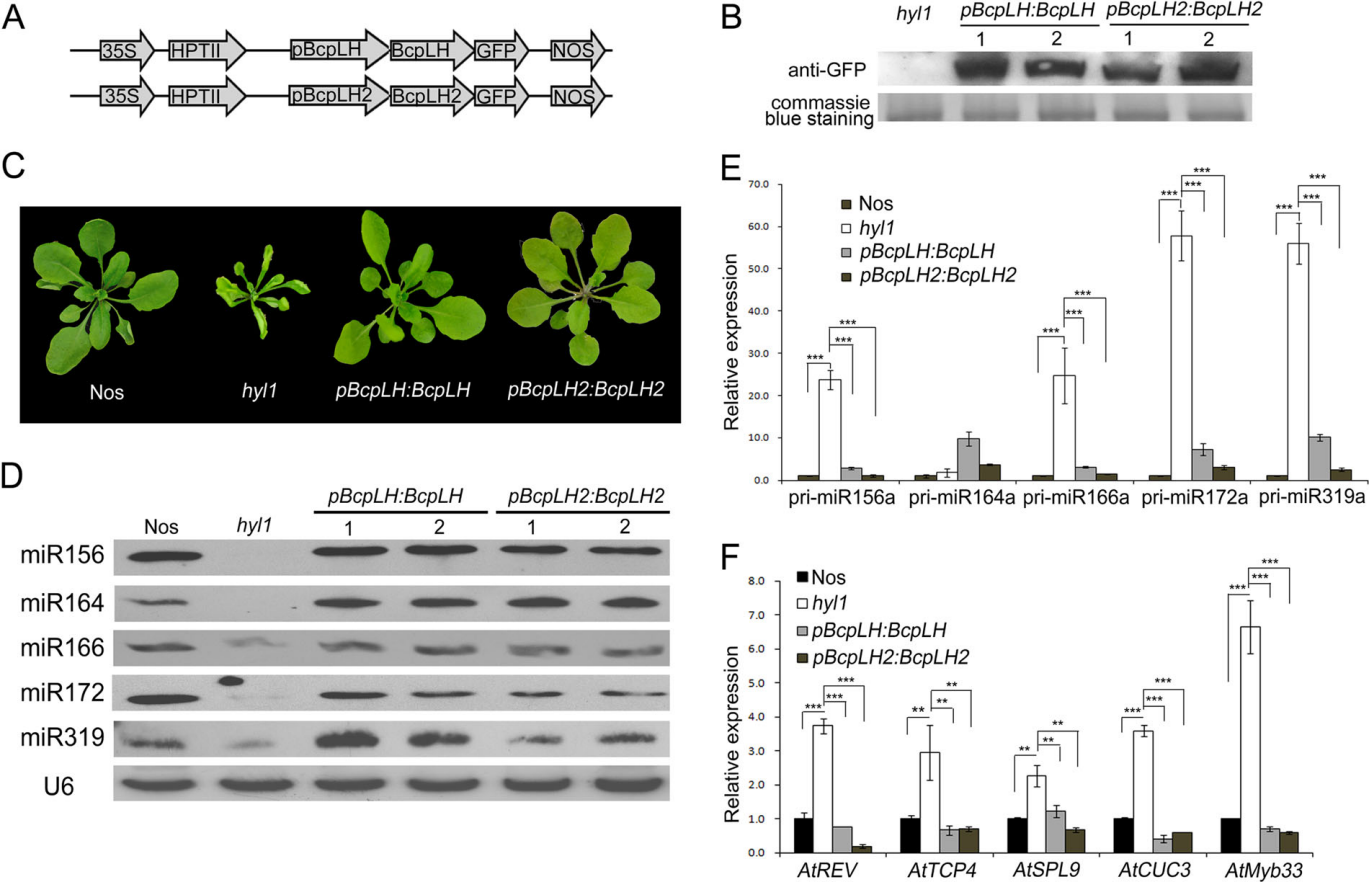
BcpLH rescued the accumulation of miRNA in hyl1. **a** Constructs of BcpLH transformed into hyl1 plants. BcpLH and BcpLH2 with GFP at the C-terminus were inserted into a pCAMBIA1301 vector under the control of pBcpLH and pBcpLH2, respectively. **b** Western blots with anti-GFP showing the BcpLH and BcpLH2 protein levels in BcpLH and BcpLH2 transgenic plants, respectively, with Coomassie staining used as a control. pBcpLH:BcpLH and pBcpLH2:BcpLH2 represent pBcpLH:BcpLH-GFP-1301/hyl1 and pBcpLH2:BcpLH2-GFP-1301/hyl1. **c** Phenotype of Nos, hyl1, pBcpLH:BcpLH and pBcpLH2:BcpLH2 plants (approximately 20 days old) in the greenhouse. **d** miRNA northern blots of BcpLH and BcpLH2 transgenic Arabidopsis. **e**, **f** Real-time PCR of pri-miRNAs and their target genes in BcpLH and BcpLH2 transgenic Arabidopsis. ACTIN expression was used as an internal control. The error bars represent the SDs calculated from three biological replicates, each of which consisted of three technical replicates. *p* < 0.05, **; *p* < 0.01, ***.

In *Arabidopsis*, *HYL1* is responsible for miRNA biogenesis. In *hyl1* mutants, the downregulation of a subset of miRNAs causes pleiotropic phenotypes, including phenotypes associated with leaf curvature, small stature and delayed phase transition^17,18^. Considering that, unlike HYL1, the BcpLH and BcpLH2 proteins lack the PPI domain, it was unclear whether *BcpLH* functions in miRNA biogenesis. Therefore, constructs of *BcpLH* and *BcpLH2* with GFP at the C-terminus under the control of *pBcpLH* and *pBcpLH2*, respectively, were introduced into the *Arabidopsis hyl1* mutant (Fig. 3a). Western blotting showed that the BcpLH and BcpLH2 proteins effectively accumulated in the transgenic lines compared with the *hyl1* mutants (Fig. 3b). The *hyl1* phenotypes were mostly rescued by *BcpLH* or *BcpLH2* in the *pBcpLH:BcpLH-GFP/hyl1* and *pBcpLH2:BcpLH2-GFP/hyl1* plants, and the degree of rescuing by *BcpLH* and *BcpLH2* was 91.7% and 89.5%, respectively (Fig. 3c, Table 2). To determine whether BcpLH and BcpLH2 contribute to miRNA biogenesis in *Arabidopsis*, northern blotting was performed, and the accumulation of miRNAs was increased in the transgenic plants compared with the *hyl1* mutants (Fig. 3d). Concomitantly, the expression of their pri-miRNAs was downregulated (Fig. 3e), and that of the corresponding miRNA-targeted genes was downregulated (Fig. 3f). We then used the *HYL1* native promoter driving *BcpLH*, *pHYL1:BcpLH-GFP*, which was subsequently transformed into the *hyl1* mutant (Supplementary Fig. 3A). The *pHYL1:BcpLH* construct expectedly rescued the phenotype of *hyl1* (Table 2, Supplementary Fig. 3B). The miRNA accumulation in the transgenic plants increased, while the expression of the corresponding target genes decreased (Supplementary Fig. 3C, D). These results suggest that BcpLH, with only two dsRNA-binding domains, is able to rescue the *hyl1* phenotype, and thus, its role in miRNA biogenesis is similar to that of HYL1.

**Table 2:** Recovery of the *hyl1* mutant in response to expression of *BcpLH* and *BcpLH2*.

	T1 total	Wild type-like	Rescue ratio
***pBcpLH:BcpLH/hyl1***	24	22	91.7%
***pBcpLH2:BcpLH2/hyl1***	19	17	89.5%
***pHYL1:BcpLH/hyl1***	21	20	95.2%

### BcpLH is a direct component of miRNA processing

**Fig. 4:**
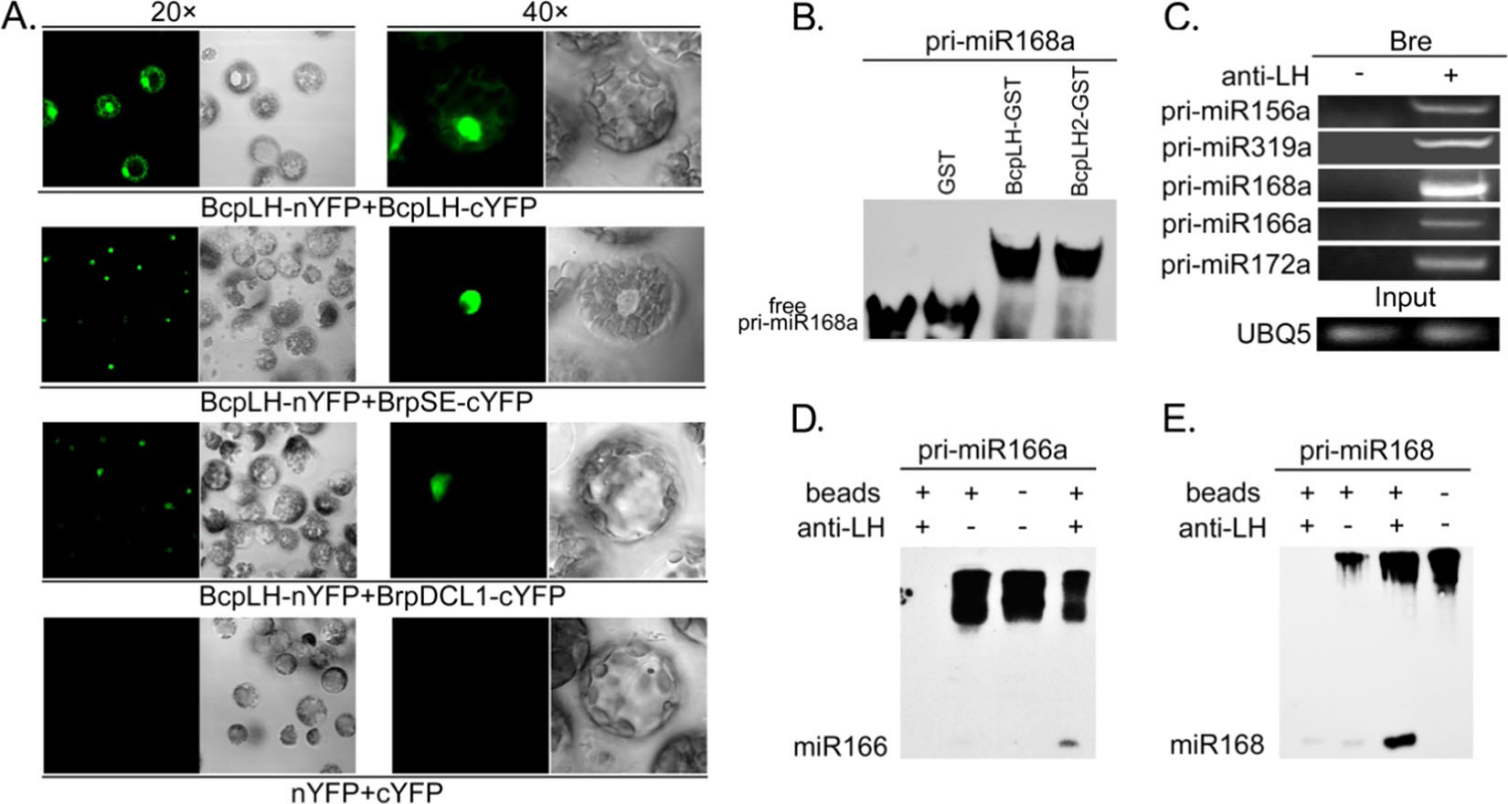
BcpLH is involved in miRNA processing. **a** BiFC showing the interactions between BcpLH and BcpLH, BrpSE, and BrpDCL1 in protoplasts of Chinese cabbage. BcpLH-nYFP and BcpLH-cYFP, BrpSE-cYFP, and BrpDCL1-cYFP were transiently coexpressed in protoplast cells of Chinese cabbage leaves. Left, YFP fluorescence. Right, bright field. **b** RNA-EMSA assay by incubating pri-miR168a with no protein, GST, BcpLH-GST or BcpLH2-GST. **c** RNA immunoprecipitation by BcpLH antibodies for pri-miRNA binding detection using wild type. Input was collected before immunoprecipitation, and UBQ5 was used as an internal control. **d**, **e** In vitro pri-miRNA processing using pri-miR166a (**d**) and pri-miR168 (**e**). pri-miRNAs were incubated with complexes immunoprecipitated with BcpLH antibodies (anti-LH) or without antibodies (beads).

*BcpLH* rescued the miRNA levels of *hyl1* in *Arabidopsis*, so we wanted to determine whether *BcpLH* participates in miRNA processing directly. In *Arabidopsis*, HYL1 colocalizes with AtSE and AtDCL1 in the nucleus^17^. The full-length CDS regions of *BrpDCL1* and *BrpSE* were amplified using cDNA synthesized from Chinese cabbage and cloned into a pEASY/blunt vector. The cloned gene fragments (*BcpLH*, *BrpDCL1*, *BrpSE1*) were sequenced and inserted into *pSAT4-nYFP* and *pSAT4-cEYFP*. Each of the two cloned constructs was transformed concurrently into the protoplasm of Chinese cabbage leaves. The results of bimolecular fluorescence complementation (BiFC) assays showed that BcpLH colocalized with BrpDCL1, BrpSE1 and BrpSE2 in the nucleus (Fig. 4a), indicating that BcpLH localizes in the D-body and is possibly associated with pri-miRNA processing in Chinese cabbage. Considering the conserved dsRNA-binding domains in BcpLH, we investigated the binding of pri-miRNAs to BcpLH by RNA electrophoresis mobility shift assays (EMSAs). Recombinant BcpLH-GST and BcpLH2-GST were expressed in *Escherichia coli* and purified by glutathione Sepharose resin. Moreover, pri-miR168a was transcribed by the T7 promoter as substrates in vitro. The purified proteins and pri-miRNAs were then incubated in mobility shift buffer at 4 °C for 2 h. Northern blotting detected the mobility shift of pri-miR168a, thus showing that pri-miRNA168a did not bind to GST but instead bound specifically to the BcpLH-GST and BcpLH2-GST proteins (Fig. 4b). Equal amounts of purified BcpLH-GST and BcpLH2-GST proteins were used for the RNA EMSA assay. As expected, the bands of the BcpLH- and BcpLH2-pri-miRNA complexes migrated more slowly than did the free pri-miRNAs, indicating the direct binding of BcpLH with the pri-miRNAs in vitro. Furthermore, RNA immunoprecipitation (RIP) was performed to investigate the binding of BcpLH with pri-miRNAs in vivo using samples from the leaves of wild-type Chinese cabbage. The pri-miRNAs loaded by the BcpLH complex were examined by RT-PCR (Fig. 4c). The tested pri-miRNAs were detected in the BcpLH complex immunoprecipitated by anti-BcpLH but not in the immunoprecipitation from the “no-antibody” controls. We concluded that pri-miRNAs bind to BcpLH proteins in Chinese cabbage plants.

It was previously reported that the efficiency of pri-miRNA processing by DCL1 was enhanced by HYL1^23,24^. In this study, we focused on the function of BcpLH in pri-miRNA processing. In conjunction with a BcpLH antibody, a co-IP complex from Chinese cabbage was used in pri-miRNA processing. The substrates for miRNA processing, pri-miR166a and pri-miR168a transcripts, were obtained in vitro under the T7 promoter. pri-miR168a was cleaved only when the BcpLH complex was added to the reaction, and mature miR166 was detected only in the presence of the BcpLH complex. In vitro miRNA processing confirmed that pri-miR168a and pri-miR166a were cleaved only in the presence of BcpLH (Fig. 4d, e). Taken together, these results suggest that BcpLH is an important and direct component in pri-miRNA processing in Chinese cabbage.

### *BcpLH* regulates leaf curvature and leafy head formation by miRNAs

**Fig. 5:**
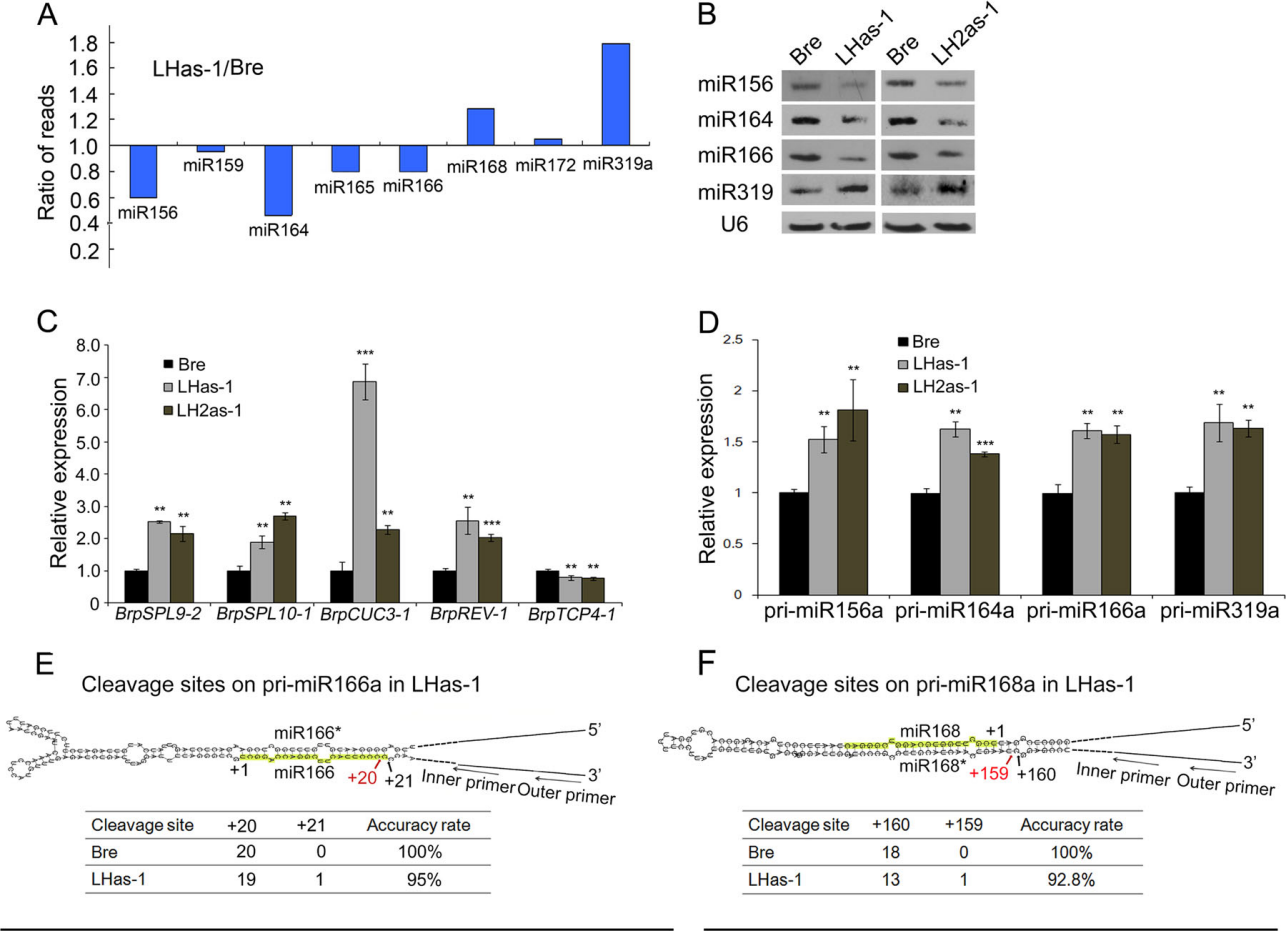
BcpLH affects the efficiency of miRNA processing in Chinese cabbage. **a** sRNA sequencing data showing the accumulation of miRNAs in Bre and LHas-1. **b** Northern blots showing the miRNA levels in Bre, LHas-1 and LH2as-1. U6 was used as an internal control. **c** Target genes and (**d**) pri-miRNAs were detected by real-time PCR in Bre, LHas-1, and LH2as-1. ACTIN expression was used as an internal control. The error bars represent the SDs calculated from three biological replicates, each of which consisted of three technical replicates. *p* < 0.05, **; *p* < 0.01, ***. **e**, **f** 5’RACE for calculating the cleavage sites in (**e**) pri-miR166a and (**f**) in pri-miR168a in Bre and LHas-1. The black arrows and red arrows indicate the correct and incorrect cleavage sites, respectively. The tables show the cleavage accuracy rates of pri-miRNAs in Bre and LHas-1.

Considering that BcpLH directly participates in miRNA processing, we suspected that the change in leaf curvature and leafy head formation in LHas-1 plants was caused by miRNAs. To examine whether knockdown of *BcpLH* affects miRNA biogenesis and the subsequent effects on Chinese cabbage, we isolated RNA samples from developing leaves (1 cm long) of the LHas-1 line at the heading stage and performed small RNA deep sequencing and RNA-seq. The abundance of a subset of miRNAs changed by more than 1.5-fold (Fig. 5a). Among the 10 miRNAs examined, miR156a-f, miR159a, miR164a, miR165a, miR165b, and miR166a-e were downregulated, while miR168a, miR172a and miR319a, and miR319b were upregulated (Supplementary Table 2). The abundance of miR156a-f decreased 1.6-fold, whereas that of miR319a and miR319b surprisingly increased 2.4-fold. Among the 21 miR156-targeted genes, 14 were upregulated, and among the 22 miR319-targeted genes, 20 were downregulated (Supplementary Table 3). The RNA-seq data showed that the expression levels of miR156-targeted *BrpSPL9-2* and miR166-targeted *BrpREV-1* were upregulated (Supplementary Table 3). Northern blotting was then performed for LHas-1 and LHas-2 to confirm the changes in miRNA accumulation in the transgenic lines (Fig. 5b). In accordance with the small RNA deep sequencing results, miR156, miR164, and miR166 decreased, while miR319 increased in LHas-1. Real-time PCR showed that the expression levels of miR156-targeted *BrpSPL9-2*, miR164-targeted *BrpCUC3-1*, and miR166-targeted *BrpREV-1* were upregulated in LHas-1 and LHas-2, which corresponded with the northern blot results and was consistent with the RNA sequencing (Fig. 5c). Corresponding to the changes in leaf curvature and leaf head formation in LHas-1, the early heading time was reminiscent of the transgenic plants harboring *pAA6:BrpSPL9-1* reported by Wang et al.^14^; the round-to-oval transition of head shape and the more wrinkled and bulging leaves were consistent with the transgenic plants harboring *pAA6:Brp-MIR319a* reported by Mao et al.^13^; and the upward curvature of the leaves is in agreement with the *pAA6:BrpREV-1* plants reported by Ren et al.^25^. These results verified that *BcpLH* regulates leaf curvature and leafy head formation via miRNAs.

To further examine whether miRNA processing efficiency or pri-miRNA cleavage accuracy or both contribute to the change in miRNAs resulting in the phenotype of LHas-1, we first quantified pri-miRNAs by real-time PCR. The results showed that the abundance of pri-miR156a, pri-miR166a and pri-miR164a increased considerably, while that of pri-miR319a decreased (Fig. 5d), thus showing that the processing of these pri-miRNAs is abolished in LHas-1 plants. The 5′ RACE (rapid amplification of cDNA ends) procedure was then performed to detect the 5′ cleavage sites in pri-miRNAs in both wild type and LHas-1. The accuracy of pri-miRNA cleavage was affected by the antisense *BcpLH*. Compared to the percentage in the wild-type pri-miR166a, 5% (1 in 20) of the cleavage sites in pri-miR166a in LHas-1 plants shifted by 1 nucleotide, and 6.7% (1 in 15) of cleavage sites in pri-miR168a shifted far away from the stem loop. Most of the cleavage sites were 16 bp away from the ssRNA-dsRNA junction of pri-miRNA in Bre. Hence, *BcpLH* is important for correct selection of the cleavage sites in pri-miRNAs (Fig. 5e, f). We conclude that *BcpLH* coordinates microRNA accumulation for the timing of leaf curvature and leafy head formation by ensuring miRNA processing efficiency or pri-miRNA cleavage accuracy in *Brassica rapa*.

### Overexpression of miRNAs partially rescued the phenotype of LHas-1

**Fig. 6:**
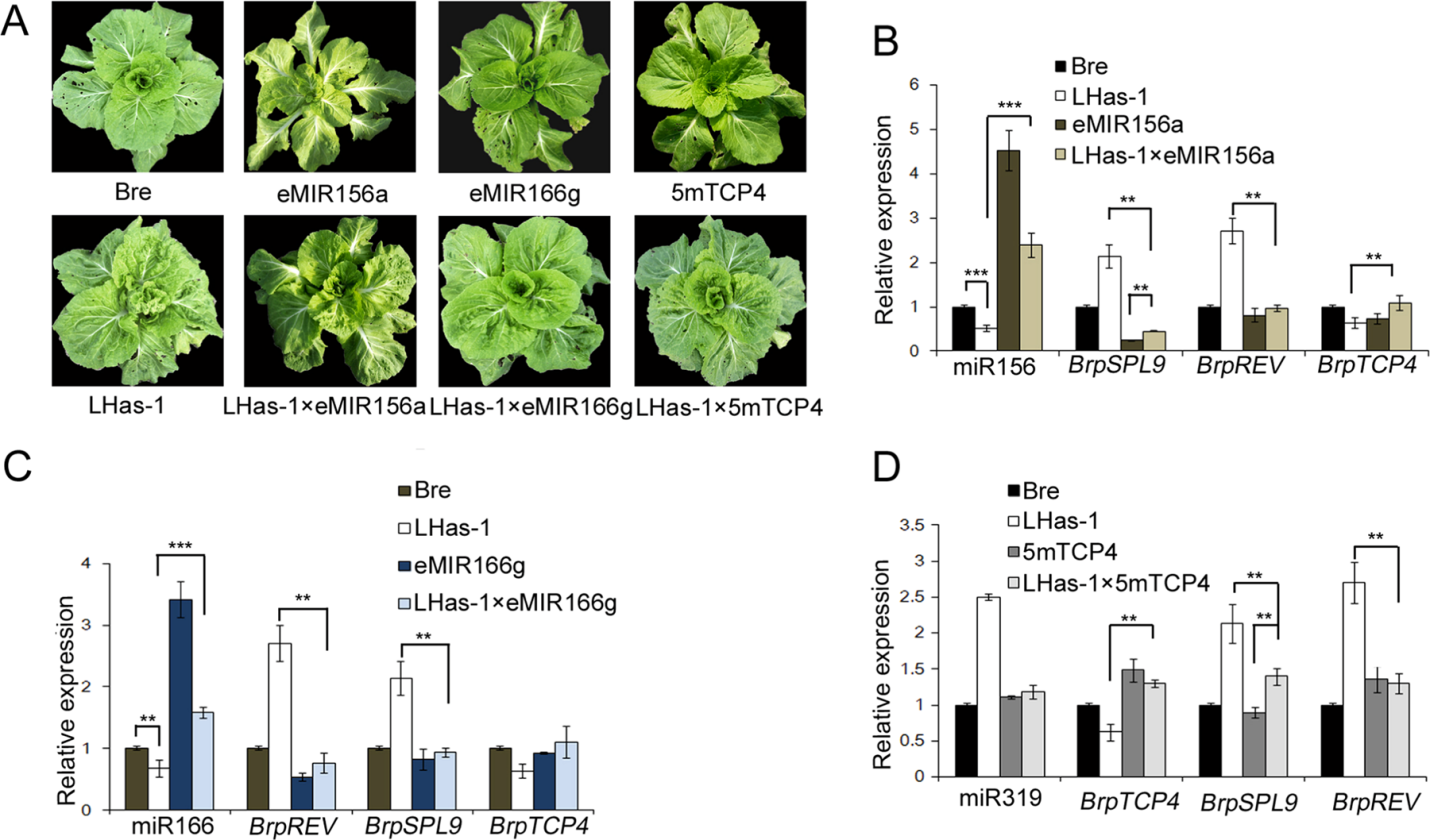
miRNA overexpression partially rescued the phenotype of LHas-1. **a** Phenotype of Bre plants, LHas-1 plants, transgenic plants and plants of transgenic crosses in the field approximately at 20 days after sowing. eMIR156a, pAA6:MIR156a/Bre; eMIR166g, pAA6:MIR166g/Bre; 5mTcp4, pAA6:5mTcp4/Bre. **b**–**d** Real-time PCR of the accumulation of miRNAs and target genes in Bre, LHas-1, eMIR156a and LHas-1 × eMIR156a (B); eMIR166g and LHas-1 × eMIR166g (**c**); and 5mTcp4 and LHas-1 × 5mTcp4 (**d**). 18 S and ACTIN were used as internal controls for miRNA and target genes, respectively. The error bars represent the SDs calculated from three biological replicates, each of which consisted of three technical replicates. *p* < 0.05, **; *p* < 0.01, ***.

miRNAs contribute to phase transition and leaf development, which cocontribute to the formation of leafy heads. As LHas-1 with altered miRNA abundances affected the timing of leaf curvature and leafy head formation, transgenic lines overexpressing miRNAs or target genes were grown to check the rescuing of LHas-1. In our study, eMIR156a miR156-overexpressing transgenic lines, with high expression of miR156 and downregulated expression of *BrpSPL9*, exhibited delayed phase transition, in which the heading time was delayed to 40 days, or no heading occurred. However, LHas-1 × eMIR156a, with upregulated miR156 and reduced expression of *BrpSPL9*, headed 35 days later than did LHas-1. eMIR166g miR166-overexpressing transgenic lines showed more downward-curved leaves, while LHas-1 × eMIR166g impaired the upward-curved leaves of LHas-1 by increased amounts of miR166 and downregulated *BrpREV* expression. In addition, 5mTCP4 transgenic plant developed more flat leaves, while LHas-1 × 5mTCP4 presented leaves with fewer wrinkles and bulges as a result of the increased expression of *BrpTCP4* (Fig. 6; Supplementary Table 4). These data showed that miRNA or target gene overexpression could partially rescue the phenotype of LHas-1, indicating the mutual effect between miRNA accumulation and mutual regulation between target genes. We concluded that *BcpLH* coordinates miR156, miR166, and miR319, causing their target genes to affect leaf development and heading characteristics. The balance among the accumulation of miRNAs and the mutual regulation of their targets thus contributes to plant development and production.

## Discussion

### BcpLH functions differently from HYL1 of Arabidopsis in the processing of some primary miRNAs

Both BcpLH and HYL1 are required for the processing of primary miRNAs and act as functional partners of DCL1 among the miRNA biogenesis machinery^26^. Although, unlike HYL1, BcpLH lacks a long PPI domain in its C-terminal region, it shows its ability to form homodimerization and ensure the correct selection of cleavage sites in pri-miRNAs. This result supports the previous findings that the N-terminal double-stranded RNA-binding domains are sufficient for processing primary miRNAs. However, the processing of some primary miRNAs is different in Chinese cabbage and Arabidopsis. In *hyl1* mutants of Arabidopsis, the miR156, miR165/6, and miR319 contents are lower than those in the wild type, concurrent with a relatively high accumulation of pri-miR156, pri-miR165/6 and pri-miR319; in Chinese cabbage, however, the miR319 content in transgenic plants with *BcpLH* antisense is much higher than that in the wild type, concurrent with a low accumulation of pri-miR319. The miR160 content is much lower in *hyl1* mutants of Arabidopsis than in the wild type, but the content in the transgenic plants of Chinese cabbage with antisense *BcpLH* is not lower than that in the wild type. This suggests that the same pri-miRNA reacts differently to BcpLH in different genetic backgrounds of Chinese cabbage and HYL1 in different genetic backgrounds of Arabidopsis.

The rosette leaves of Chinese cabbage and Arabidopsis are essentially flat. However, the rosette leaves of *hyl1* mutants and LHas-1 plants are upward curving in the transverse direction, possibly due to the reduced contents of miR156 and miR165/6. The difference is that the wrinkled, bulging and wavy margins typical of *jaw-1* mutants (which present enhanced expression of miR319a) occur on the rosette leaves of LHas-1 plants but do not occur on the rosette leaves of *hyl1* mutants. The enhanced expression of miR319a in the rosette leaves of LHas-1 plants causes the wrinkled, bulging and wavy margins of the leaves. We suggest that the wrinkled, bulging and wavy margin leaf phenotype caused by BcpLH occurs mainly through miR319a.

Recently, we found that several pri-miRNAs bind to BcpLH differentially, and they compete with each other for binding ability. In LHas-1 plants, the disruption of the original balance between these miRNAs may alter the competence of some pri-miRNAs, thereby altering the levels of the related miRNAs. An attempt has been made to identify whether the enhanced expression of miR319a in the rosette leaves of LHas-1 plants is caused by the reduced levels of miR165/6 and/or miR156.

### BcpLH regulates the direction, degree, and timing of leaf curvature

Plants have formed a set of mechanisms to coordinate the morphogenesis of organs and the timing of developmental events. The flat leaves of Chinese cabbage generated at the seedling and rosette stages produce enough photosynthetic products to support plant growth, whereas those at the folding and heading stages are upwardly and inwardly curved for nutrient storage. The coordination of morphological changes and phase transition ensures the formation of leaf curvature. This coordination is apparently disrupted by antisense *BcpLH*. In LHas-1 plants, silencing of *BcpLH* via antisense causes rosette leaves to transition from being flat to being upward and causes the folding-stage leaves to transition from being upward to curving inward; moreover, the degree of upward curvature increases, and the inward curvature occurs sooner compared with that of wild type. Moreover, the wrinkled, bulging and wavy margins occur on rosette leaves. These altered leaf characteristics are beneficial for early heading, as LHas-1 plants form leafy heads much sooner than do wild-type plants.

The changes in the direction, degree and timing of leaf curvature are attributable to a decrease in a subset of miRNAs. In LHas-1 plants, miR165/6, miR156, and miR164 accumulation decreases; miR168, miR172, miR319 accumulation increases; and the accumulation of many of the other miRNAs does not change considerably. miR165/6, miR156, and miR319 regulate the development of adaxial identity, phase transition and arrest cell division at the front of leaves, respectively. Therefore, it is important to analyze the relationships between these miRNAs and the timing of leaf curvature.

### BcpLH coordinates miR165 with miR156 and miR319 for the timing of leaf curvature

In Chinese cabbage, miR156 prolongs the juvenile phase and delays the adult phase by silencing *BrpSPL9-2*, leading to early leaf incurvature and heading^14^; miR319a regulates differential cell division arrest in forward leaf regions by silencing the *BrpTCP4* gene, resulting in wrinkled, bulging and wavy leaf margins and causing the head shape to transition from round to cylindrical^13^; and miR166 regulates the direction of leaf incurvature, causing changes in head size and heading time^25^. BcpLH and BcpLH2 control the expression levels of the *BrpSPL9-2*, *BrpREV*, and *BrpTCP4* genes via miR156, miR166 and miR319, respectively, and thus, their downregulation affects the phase transition and head shape simultaneously. As such, miR165/6 coordinates with miR156 and miR319 to determine the timing of leaf curvature. The balance between the relative abundance of these miRNAs is essential for the correct timing of leaf curvature during vegetative growth. In Arabidopsis, HYL1 regulates leaf flatness by modulating the ratio of the expression of genes involved in adaxial to abaxial characteristics (adaxial to abaxial ratio), which determines the direction and extent of leaf incurvature^27^. In Chinese cabbage, BcpLH regulates the balance among miR156, miR165/6 and miR319 and ensures that leaf curvature occurs at the proper time during vegetative growth.

BcpLH promotes the processing of pri-miR165/6 and pri-miR156 but inhibits the processing of pri-miR319a. The contents of miR156, miR165/6 and miR319 affect the expression levels of HD-ZIP III, *SPL*, and *TCP* genes, respectively, and influence the adaxial identity, phase transition and cell division arrest at the front of leaves, thus affecting the timing of leaf curvature. Furthermore, the balance among miR156, miR165/6 and miR319 under the control of BcpLH facilitates the formation of leafy heads through the correct timing of leaf curvature. Hence, BcpLH has the potential for genetic manipulation of agricultural products. In future research, we could alter the expression of *BcpLH* and *BcpLH2* to generate plants with characteristics such as better or more effective storage, which would bring great benefits to agricultural production.

## Methods

### Plant materials and growth conditions

An inbred line of Chinese cabbage (*B. rapa* ssp*. pekinensis* cv. Bre) was used in this study. The seeds were sown in a greenhouse on August 8, 2014. Two weeks later, the seedlings were transplanted into the field at the Songjiang Farm Station of SIPPE in early September.

The *in planta* transformation procedures with Bre using the vernalization-infiltration method are described by Bai et al.^22^. Briefly, Brassica plants with small flower buds at the early bolting stage were used for transformation. The plants were placed upside down in a vacuum desiccator that contained both infiltration media and the engineered *Agrobacterium* for vacuum infiltration. The *Agrobacterium*-infected plants were then grown in a dark room and incubated at 22/18 °C, after which they were transferred to a chamber room after 2 days. The pollen of the Bre plants was then used to pollinate the transformed flowers manually. The seeds of the transgenic plants were harvested after they plants grew for 1–2 months in a growth chamber.

*Arabidopsis thaliana* wild-type plants and *hyl1* (Nossen ecotype) mutants were used in this study. The growth conditions and transgenic methods are described by Wu et al.^19^.

### RNA analysis

For isolating total RNA from plant samples, 1 ml of TRIzol per 0.2 g of plant tissue was used for extraction, and phenol:chloroform:isoamyl alcohol and chloroform were added for phase separation, followed by ethanol precipitation.

Nothern blotting was performed as described previously by Wu et al.^19^. Briefly, 30 µg of total RNA was loaded onto a 19% PAGE gel for electrophoresis at 150 V for 4 h, after which the gels were transferred to a Hybond membrane (Amersham Biosciences, GE Healthcare) subjected to 200 mA for 2 h. After UV cross-linking was performed, the membrane was then hybridized in ULTRAhyb^®^ Ultrasensitive Hybridizaton Buffer (Ambion, Austin, TX, USA) with DNA oligo probes. The probes were in the antisense orientation to the mature miRNA or U6 transcripts, with biotin labels at their 3′ terminal (TaKaRa, Otsu, Japan). The northern blot results were generated with a Light Shift EMSA Kit (Thermo Scientific, Waltham, MA, USA) and imaged using a FLA-5000 Phosphor imager (Fujifilm).

Total RNA samples were extracted from plant leaves using TRIzol, extracted with phenol:chloroform:isoamyl alcohol and chloroform, and then precipitated with ethanol.

For northern blotting, 30 µg of total RNA was resolved by 19% PAGE electrophoresis in 1 × TBE buffer and then transferred to a Hybond membrane (Amersham Biosciences, GE Healthcare), which was subjected to 200 mA for 2 h. The UV cross-linked membrane was subsequently hybridized in ULTRAhyb^®^ Ultrasensitive Hybridizaton Buffer (Ambion, Austin, TX, USA) using antisense probes of 3′-biotin-labeled oligo DNA (TaKaRa, Otsu, Japan) to mature miRNA or U6 transcripts. The hybridization signals were developed with a Light Shift EMSA Kit (Thermo Scientific, Waltham, MA, USA) and imaged using a FLA-5000 Phosphor imager (Fujifilm).

To perform quantitative real-time PCR, 50 μg of RNA was treated with DNase I (TaKaRa) to remove DNA contamination, followed by RNA extraction with phenol:chloroform. Five micrograms of RNA was reverse transcribed to produce cDNAs with PrimeScript^®^ Reverse Transcriptase (TaKaRa) in conjunction with oligo(dT) primers. Real-time PCR was performed with specific primer pairs (Supplementary Table 5) in a MyiQ2 Two-color Real-time PCR Detection System (Bio-Rad, Richmond, CA, USA). At least 3 biological replicates of quantitative PCR were performed for each gene. The relative transcript level of each gene was normalized to that of ACTIN cDNA for quantitation.

### Protein analysis

Anti-GFP (Sigma-Aldrich, St Louis, MO, USA; F3165, 1:5000 dilution), anti-GST (Sigma-Aldrich; 1:5000 dilution), anti-LH (NEB, 1:3000 dilution) and anti-HYL1 (Agrisera, 1:1000 dilution) antibodies were used for Western blotting. The secondary antibodies used were goat-developed anti-rabbit IgG antibodies (GE Healthcare; NA931V, 1:20 000 dilution).

### BiFC assay

Paired constructs were coexpressed in *Arabidopsis* protoplasts for 12 h at 22.5 °C in the dark and subjected to confocal microscopy (Zeiss LSM 510 Meta) for imaging. BiFC signals were excited at 658 nm and detected with a narrow barrier filter.

### RIP

Leaf tissue from five-week-old transgenic *Arabidopsis* plants was ground under liquid nitrogen and homogenized in 5 mL/g lysis buffer [50 mM Tris-HCl (pH 7.4), 100 mM KCl, 2.5 mM MgCl_2_, 0.1% NP-40, and 2× complete protease inhibitor cocktail; Roche]. After centrifugation for 15 min at 9500 × g, the clarified lysate was precleared for 20 min at 4 °C with 10 μL of bed volume protein A-agarose (30 μg protein A)/milliliter. The precleared lysates were reacted with 4 μg of anti-LH (NEB) or anti-GFP (Sigma-Aldrich)/milliliter for 1 h at 4 °C and then with 50 μL of bed volume protein A-agarose (150 μg protein A)/milliliter for 3 h at 4 °C. The precipitates were washed three times in lysis buffer and then divided for protein and RNA analyses. Nucleic acids were recovered by treatment with 3 volumes of proteinase K solution [100 mM Tris-HCl (pH 7.4), 10 mM EDTA, 150 mM NaCl, 2% SDS, and 0.2 μg/μL proteinase K] for 15 min at 65 °C, extracted with saturated phenol and phenol:chloroform, and then precipitated with ethanol. Five micrograms of RNA from the input extract or from IP fractions representing 150 mg of tissue was used for qPCR analysis. UBQ5 was used as a control.

### RNA-EMSAs

BcpLH and BcpLH2 were independently cloned in frame in a pGEX4T-1 vector for bacterial expression and purification of fusion proteins, which were then used in RNA-EMSA assays. Pre-miR168a stem-loop RNA was transcribed by the T7 promoter in vitro. One microgram of purified GST, BcpLH-GST and BcpLH2-GST protein each was then incubated with the pre-miRNAs (1.5 pmol). The reactions were incubated in binding buffer [20 mM HEPES-KOH (pH 7.5), 10 mM KCl, 20 mM MgCl_2_, 0.5 mM EDTA, and 0.5 mM DTT] at 4 °C for 30 min and were subsequently resolved on a 4.5% nondenaturing glycerol polyacrylamide gel, after which they were exposed to a storage phosphor imager screen for the detection of biotin-labeled miRNA.

### Pri-miRNA processing in vitro

RNA substrates were transcribed under the T7 promoter in vitro using PCR-generated templates. The in vitro transcription of RNAs was carried out for 3 h or overnight at 37 °C in one reaction containing 1 µL of DNA template (100 ng), 4 µL of 5 × transcription buffer [400 mM HEPES (pH 7.5), 10 mM spermidine, 200 mM DTT, 125 mM MgCl_2_ and each dNTP at 20 mM], 1 µL of RNase inhibitor (Ambion), 2 µL of T7 RNA polymerase and 12 µL of water. DNase-treated RNA was fractionated on a 6% polyacrylamide and 8 M urea gel (denaturing gel) and eluted overnight from gel slices in RNA elution buffer [0.3 M NaAc (pH 5.5) and 2% SDS] using a Thermomixer R (Eppendorf) at 4 °C under 1200 rpm; afterward, the RNA was precipitated with ethanol and stored in RNase-free water. Briefly, 10 µL of each RNA cleavage assay mixture contained 20 mM Tris-HCl (pH 7.0), 50 mM NaCl, 4 mM MgCl_2_, 5 mM ATP, 1 mM GTP, 2 units of RNase inhibitor (TaKaRa), RNA substrate, and the co-IP protein complex with anti-LH. After incubation at 37 °C for 30 min, the products were extracted with phenol:chloroform and precipitated. The processed products were fractionated by PAGE in a 19% acrylamide urea gel and detected by northern blotting.

## Supplementary information

Table S1

Table S2

Table S3

Table S4

Figures
