# Supplementary material for: BcpLH organizes a specific subset of microRNAs to form a leafy head in Chinese cabbage (Brassica rapa ssp. pekinensis)
Source: Hortic Res. 2020 Jan 1;7:1. doi: 10.1038/s41438-019-0222-7 (PMC6938484; doi:10.1038/s41438-019-0222-7)
Supplement: Supplementary file 1 — Table S1 [file 41438_2019_222_MOESM1_ESM.docx]

**Supplementary Table 1. Leaf formation of the transgenic plants with *BcpLH* antisense (LHas-1) at four stages.** The seeds were sowed in pots and grown at 22℃ in SIPPE Phytotron. The seedlings were transferred to the field on August 24, 2012 on SIPPE Farm Station. Number of plants for each measurement was more than 20. The data were the mean of 20 leaves. ND, not detected. B/P, blade-to-petiole; L/W, length-to-width; -, no; +, few; ++, medium; +++, many.

| Leaf parameters | WT | LHas-1 |  |
| --- | --- | --- | --- |
| *Seedling stage (leaf 4)* | | | |
| Blade base angle (^o^) | 123.9±2.56 | 120.3±3.41 |  |
| Blade length cm) | 5.7±0.59 | 5.6±0.64 |  |
| Blade width (cm) | 3.9±0.62 | 4.2±0.52 |  |
| Blade L/W ratio | 1.5±0.13 | 1.4±0.10 |  |
| B/P length ratio | 5.0±0.46 | 4.9±0.45 |  |
| Bulges | - | - |  |
| Hydathodes | 25.8±1.45 | 26.6±1.53 |  |
| Leaf Lobes | - | - |  |
| Petiole length cm) | 1.1±0.08 | 1.2±0.10 |  |
| Petiole width | ND | ND |  |
| Petiole L/W ratio | ND | ND |  |
| Wrinkled leaves | - | - |  |
| *Rosette stage (leaf 8)* | | | |
| Blade base angle (^o^) | 123.88±2.40 | 119.15±3.31 |  |
| Blade length cm) | 10.65±0.60 | 10.27±0.51 |  |
| Blade width (cm) | 8.12±0.58 | 8.21±0.45 |  |
| Blade L/W ratio | 1.31±0.09 | 1.25±0.08 |  |
| B/P length ratio | 4.75±0.39 | 4.87±0.41 |  |
| Bulges | - | - |  |
| Hydathodes | 81.52±1.64 | 86.52±4.12 |  |
| Leaf Lobes | - | - |  |
| Petiole length cm) | 2.26±0.25 | 2.12±0.22 |  |
| Petiole width | 0.5 | 0.5 |  |
| Petiole L/W ratio | 4.52±0.51 | 4.24±0.43 |  |
| Wrinkled leaves | - | + |  |
| *Folding stage (leaf 14)* | | | |
| Blade base angle (^o^) | 99.76±2.47 | 97.16±3.63 |  |
| Blade length cm) | 20.12±1.33 | 20.8±1.65 |  |
| Blade width (cm) | 19.08±1.80 | 19.84±1.86 |  |
| Blade L/W ratio | 1.05±0.06 | 1.05±0.05 |  |
| B/P length ratio | 4.51±0.34 | 4.58±0.42 |  |
| Bulges | - | + |  |
| Hydathodes | 97.56±2.48 | 98.24±2.75 |  |
| Leaf Lobes | + | + |  |
| Petiole length cm) | 4.48±0.37 | 4.56±0.42 |  |
| Petiole width | 3 | 3 |  |
| Petiole L/W ratio | 1.49±0.12 | 1.52±0.14 |  |
| Wrinkled leaves | + | ++ |  |
| *Heading stage (leaf 18)* | | | |
| Blade base angle (^o^) | 89.76±2.47 | 89.44±2.53 |  |
| Blade length cm) | 21±1.25 | 21.44±1.32 |  |
| Blade width (cm) | 23±1.52 | 23.88±1.51 |  |
| Blade L/W ratio | 0.91±0.05 | 0.90±0.04 |  |
| B/P length ratio | 5.43±0.46 | 5.62±0.38 |  |
| Bulges | + | + |  |
| Hydathodes | 116.04±5.13 | 115.84±5.01 |  |
| Leaf Lobes | + | + |  |
| Petiole length cm) | 3.88±0.22 | 3.82±0.24 |  |
| Petiole width | 4 | 4 |  |
| Petiole L/W ratio | 0.97±0.05 | 0.95±0.06 |  |
| Wrinkled leaves | ++ | +++ |  |
