# Supplementary material for: BcpLH organizes a specific subset of microRNAs to form a leafy head in Chinese cabbage (Brassica rapa ssp. pekinensis)
Source: Hortic Res. 2020 Jan 1;7:1. doi: 10.1038/s41438-019-0222-7 (PMC6938484; doi:10.1038/s41438-019-0222-7)
Supplement: Supplementary file 4 — Table S4 [file 41438_2019_222_MOESM4_ESM.docx]

**Supplemental Table 4. Primers used in this study**

| **Primer name** | | **Sequence（5’-3’）** | | | | | |
| --- | --- | --- | --- | --- | --- | --- | --- |
| **Binary vector construction** | | | | | | | |
| BcpLH-1S-SalI | | ACGCGTCGACATGACGGCGAATGAAGTTTCC | | | | | |
| BcpLH-822A-BamHI | | CGGGATCCTGCTTGGCTTGCTTCTGTCTCC | | | | | |
| BcpLH2-1S-SalI | | ACGCGTCGACATGACTGCGAATGAAGTTCCC | | | | | |
| BcpLH2-834A-BamHI | | CGGGATCCTGCTAAGTTTGCTTCTGTCTCC | | | | | |
| BcpLH2-1S-BamHI | | CGGGATCCATGACTGCGAATGAAGTTCCC | | | | | |
| BcpLH2-837A-SalI | | ACGCGTCGACTTATGCTAAGTTTGCTTCTGTCTCC | | | | | |
| BrpDCL1-1S-XmaI | | TCCCCCCGGGATGGTAATGGAGGATGAGCGTAG | | | | | |
| BrpDCL1-5541A-SalI | | ACGCGTCGACTCAAGAATAAGTTTTATTAAGAAG | | | | | |
| BrpSE1-1S-XmaI | | TCCCCCCGGGATGGCCGACGAGACTCTTCCTCC | | | | | |
| BrpSE1-2133A-XhoI | | CCGCTCGAGCTACAAGCTCCTGTAATCAATA | | | | | |
| BrpSE2-1S-XmaI | | TCCCCCCGGGATGGCCGATGTGACGCTCCCTCC | | | | | |
| BrpSE2-2079A-XhoI | | CCGCTCGAGCTACAAGCTCCTATAATCAATA | | | | | |
| BcpLH-1S-KpnI | | GGGGTACCATGACGGCGAATGAAGTTTCC | | | | | |
| **Real-time PCR** | | | | | | | |
| ACTIN-S  ACTIN-A | | | | TGGCATCAYACTTTCTACAA  CCACCACTDAGCACAATGTT | | | |
| AtTCP4-RT-S  AtTCP4-RT-A | | | | AGGGTTTCTGTTCGCTCCTCCTAC  GTCGGTGGAGATGGATTGGTGAT | | | |
| AtPHB-RT-S  AtPHB-RT-A | | | | TTTCTATAGCAGAGGAGGCCC  AGGAGCATACATCTGCGTGT | | | |
| AtREV-RT-S  AtREV-RT-A | | | | TGCTCCACTTGTTCCCTC  TAGCCTTACGACCCGATT | | | |
| AtSPL9-RT-S  AtSPL9-RT-A | | | | CAAGTGGAAGGTTGTGGGATG  GTCGCCAATTCCCTTGTAGCT | | | |
| AtHYL1-RT-1S  AtHYL1-RT-287A | | | | ATGACCTCCACTGATGTTTCCTC  ACAGGTTGTGAAACACATTGGC | | | |
| Atpri-miR166a-S38 | | | | CCTTCACATTTCAGATTTGATTAGGG | | |  |
| Atpri-miR166a-311A | | | | | AGCAATGTAGAAAAGTTCA |  |  |
| Atpri-miR156a-S45 | | | | | TCTCTGAAGTTGGACTAATTGTGAA |  |  |
| Atpri-miR156a-283A | | | | | ATTAAAGGCTAAAGGTCTCCTCCC |  |  |
| Atpri-miR319a-RT-2S | | | | | GCTTCCTTGAGTCCATTCACA |  |  |
| Atpri-miR319a-RT-2A | | | | | GCTCCCTTCAGTCCAATCAAA |  |  |
| AtCUC3-RT-S | | | | | ACTGCTGGATACTGGAAA |  |  |
| AtCUC3-RT-A | | | | | GAGACGACAGGGTTGATT |  |  |
| AtMYB33-RT-1214S | | | | | TCGTCATCTCCTCCACACTCTG |  |  |
| AtMYB33-RT-1460A | | | | | CCTCGGATTTAGTTTGGGATAC |  |  |
| AtMYB65-RT-716S | | | | | CTTCCCCAAAGCAAATCTG |  |  |
| AtMYB65-RT-974AA | | | | | TTCACTGCCCCAAACAAG |  |  |
| AtARF10-RT-1S | | | | | CCTTCAGGTAGCTTGGGACGA | |  |
| AtARF10-RT-1A | | | | | GATGATGCCTCGGGTTGAAAC | |  |
| BcpLH-RT-645S | | | | CAAGAAGAAGGCTCGGAAAGGA | | | |
| BcpLH-RT-832A | | | | GAGACAGCTATGCTTGGCTTG | | | |
| BcpLH2-RT-S40 | | | | CTTCTCCTCGGTTTCCAATTA | | | |
| BcpLH2-RT-626S | | | | CAAGGAGGGCTCAGTTCAAG | | | |
| BcpLH2-RT-860A | | | | TCACACACTCACAGAACACAGAC | | | |
| ATHYL1-RT-1063S | | | | AGTGTGGAGACAGAGAAAATTGAG | | | |
| ATHYL1-RT-1232A | | | | GCGTGGCTTGCTTCTGTCTCC | | | |
| BrpTCP4-1-RT-784S | | | | CAGCCAGTACTTGGCCAAAG | | | |
| BrpTCP4-1-RT-870A | | | | ATCAAACCAAGCACGGATCAT | | | |
| BrpAGO1-1-RT-162S | | | | TCAGTCTCAACAGGGAGGTCG | | | |
| BrpAGO1-1-RT-447A | | | | CAGCTCGGGAACTGATTGTCT | | | |
| BrpREV-1-2S | | | | TAGGAAGACAGCCTGCTGTTT | | | |
| BrpREV-1-2A | | | | ACACCTCCAAGGAAGGAAAGA | | | |
| BrpSPL9-1-592s-RT | | | | CCAGTGGCAACACCATCA | | | |
| BrpSPL9-1-982a-RT | | | | GAACTCACCCATTGTCGT | | | |
| BrpSPL9-2-589s-RT | | | | ATGAGACGGCCACCGTC | | | |
| BrpSPL9-2-996a-RT | | | | AGTCGTTCCACCGCTTATC | | | |
| BrpSPL10-1-788s-RT | | | | ATGTATGGAGTCCCAGTTACG | | | |
| BrpSPL10-1-1194a-RT | | | | CGAATAGAAACACTCCCATTAGGT | | | |
| Brppri-miR168a-RT-S3 | | | | GAYTCGCTTGGTGCAGGTC | | | |
| Brppri-miR168a-RT-82A | | | | GGAKCCRATCCCTGCTCAC | | | |
| Brppri-miR156a-RT-S37 | | | | GAGTGAATGAGYTGGGRCAA | | | |
| Brppri-miR156a-RT-78A | | | | CAGAAAGAGCAGTGAGCACGC | | | |
| Brppri-miR319a-RT-S105 | | | | GYTTCCGACTCATYCATCCAAA | | | |
| Brppri-miR319a-RT-20A | | | | RGGAGCTCCCTTCAGTCCAA | | | |
| BrpCUC2-1-RT2-602S | | | | TCTCCACAAACTACCGTGAACA | | | |
| BrpCUC2-1-RT2-826A | | | | TGAATGAGTCGACGTCAACATC | | | |
| BrpARF16-1-1329S | | | | TCAGCATCCGGATTACAACAA | | | |
| BrpARF16-1-1589A | | | | CTATCGATGTTTCGCAACCCTA | | | |
| BrpMYB65-1-836S | | | | ACGGAAACCAACATCCATCTGA | | | |
| BrpMYB65-1-1212A | | | | ATTTTCACCCGCACTAAGTTGC | | | |
| **5' RACE** | | | | | | | |
| BrpMIR166a-198A | | | | | CGGTCCCAAAAGACCAAAAAATTA | | |
| BrpMIR166a-170A | | | | | AATGTAAGGATTGATCAATG | | |
| BrpMIR168a-271A | | | | | CACGATAAAGATTTTAACTTTTAGCC | | |
| BrpMIR168a-233A | | | | | AACAGAGATTGAAGAAGATATG | | |
| **RNA transcription** | | | | | | | |
| BrpMIR166a-S131 | | | TAATACGACTCACTATAGGGAGAGAGGGAAGGGGCTTTCTCTTTTGAGG | | | | |
| BrpMIR166a-43A | | | GTAAGCAGGGAGCAATAATTGG | | | | |
| BrpMIR156a-S11 | | | TAATACGACTCACTATAGGGAGACACACAGAAACTGACAGAAGAGA | | | | |
| BrpMIR156a-97A | | | GATCAGCACGGGAATCTGACAGAAAG | | | | |
| BrpMIR319a-S23 | | | TAATACGACTCACTATAGGGAGATTGTAGGGATATATATCGAGGGA | | | | |
| BrpMIR319a-97A | | | AAGAGAGAGGGAGCTCCCTTCAGTCC | | | | |
| BrpMIR168a-S54 | | | TAATACGACTCACTATAGGGAGATTCAGATTTGAAGAGACAGAGAGGT | | | | |
| BrpMIR168a-145A | | | CTGTTTGGGTTTTTGCTGGTTTTCAC | | | | |
| **Primers for sequencing** | | | | | | | |
| pJR1-5S-1 | | | GGAGAGGACAGGGTACC | | | | |
| pCAMBIA-lacZ-A | | | TGCTGCAAGGCGATTAAGTTGGGT | | | | |
| T7-1S | | | TAATACGACTCACTATAGGG | | | | |
| pSAT4-5'UTR-1165S | | | GGACGTCGAGAGTTCTCAACACAACA | | | | |
| pSAT4-NOS-A | | | GAGATAGATTTGTAGAGAGAGACTGG | | | | |
| **Probes for Northern blotting** | | | | | | | |
| anti-miR166-biotin | | | GGGGAATGAAGCCTGGTCCGA | | | | |
| anti-miR167-botin | | | TAGATCATGCTGGCAGCTTCA | | | | |
| anti-miR168-biotin | | | TTCCCGACCTGCACCAAGCGA | | | | |
| anti-miR159-biotin | | | TAGAGCTCCCTTCAATCCAAA | | | | |
| anti-miR319-biotin | | | AGGGAGCTCCCTTCAGTCCAA | | | | |
| anti-miR156-biotin | | | GTGCTCACTCTCTTCTGTCA | | | | |
| anti-miR172-biotin | | | ATGCAGCATCATCAAGATTCT | | | | |
| anti-miR164-biotin | | | TGCACGTGCCCTGCTTCTCCA | | | | |
| anti-U6-biotin | | | TCATCCTTGCGCAGGGGCCA | | | | |
