# Supplementary material for: BcpLH organizes a specific subset of microRNAs to form a leafy head in Chinese cabbage (Brassica rapa ssp. pekinensis)
Source: Hortic Res. 2020 Jan 1;7:1. doi: 10.1038/s41438-019-0222-7 (PMC6938484; doi:10.1038/s41438-019-0222-7)
Supplement: Supplementary file 5 — Figures [file 41438_2019_222_MOESM5_ESM.docx]

**
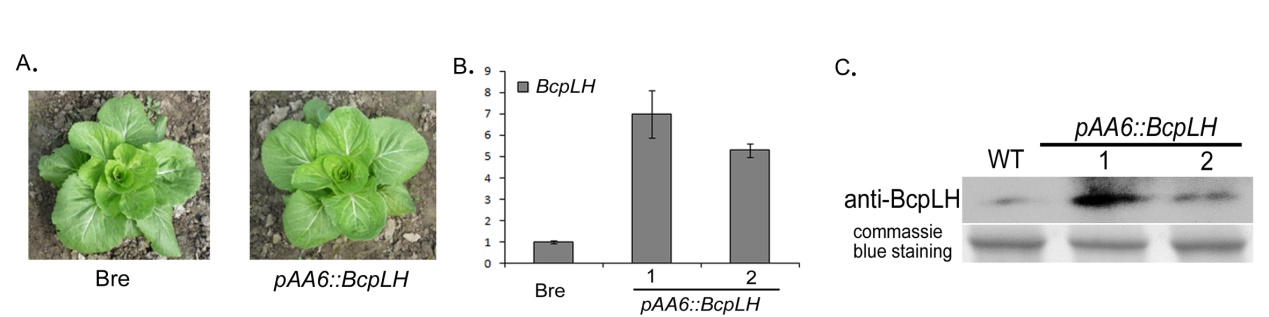
**

**Supplemental Figure 1.** **Overexpression of *BcpLH* in Chinese cabbage brings no change.** (A) Phenotype of *BcpLH* transgenic plants in the field. (B) The mRNA levels of *BcpLH* in Chinese cabbage transgenic plants by realtime-PCR. *ACTIN* was used as internal control. Error bars represent SD calculated from three biological replicates, each of which had three technical replicates. (C) The protein levels of BcpLH in Chinese cabbage transgenic plants by western blotting. Commassie staining was used as internal control.


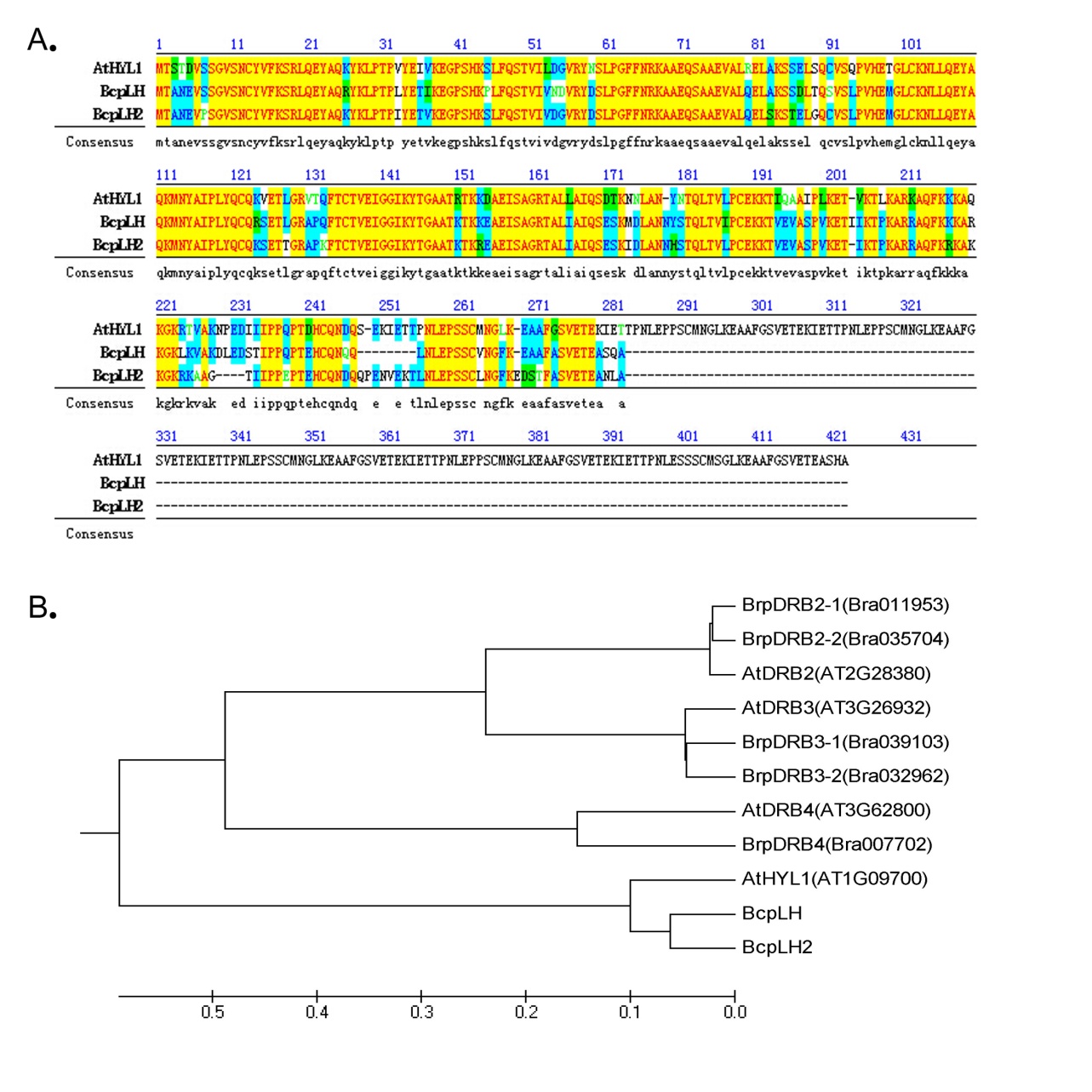


**Supplemental Figure 2. *BcpLH* and *BcpLH2* are the homologous genes of *AtHYL1*.**

(a) Multiple sequence alignment of *AtHYL1* and *BcpLH*, *BcpLH2* gene sequences. (b) The phylogenetic tree of AtDRBs and BrpDRBs. MEGA 5 program was used for clustal alignment of the sequences and construction of phylogenetic tree.


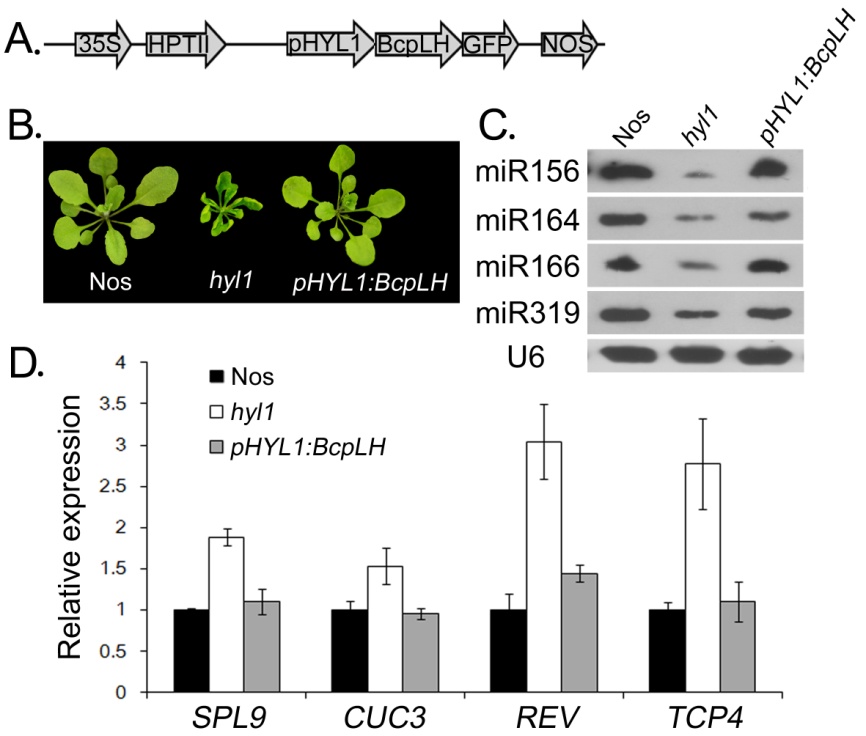


**Supplemental Figure 3. *BcpLH* under *HYL1* native promoter rescued the phenotype of *hyl1*.**

(A) Constructs of *BcpLH* transformed into *hyl1* plants. *BcpLH* with *GFP* at C terminal were inserted to *pCambia1301* under *pHYL1*. (B) Phenotype of Nos, *hyl1*, *pHYL1:BcpLH* about 20 days in green house. (C) miRNA northern blot in *BcpLH* and *BcpLH2* transgenic Arabidopsis. (D) Real-time PCR for target genes in *BcpLH* transgenic Arabidopsis. *ACTIN* expression was used as an internal control. Error bars represent SD calculated from three biological replicates, each of which had three technical replicates.
